# Supplementary material for: From sequence to enzyme mechanism using multi-label machine learning
Source: BMC Bioinformatics. 2014 May 19;15:150. doi: 10.1186/1471-2105-15-150 (PMC4229970; doi:10.1186/1471-2105-15-150)
Supplement: Additional file 2 — Java code of ml2db. Additional file ml2db_code.tar.gz contains the Java source code to run the multi-label machine learning experiments and save the results to database. The code’s Javadoc is included. [file 1471-2105-15-150-S2.zip › additional file 2/ml2db/ml2db/doc/main/ml2db/al/learning/class-use/ExperimentDetails.html]

Uses of Class main.ml2db.al.learning.ExperimentDetails


JavaScript is disabled on your browser.


- Overview
- Package
- Class
- Use
- Tree
- Deprecated
- Index
- Help

- Prev
- Next

- Frames
- No Frames

- All Classes

## Uses of Class main.ml2db.al.learning.ExperimentDetails

- Packages that use ExperimentDetails

  | Package | Description |
  | --- | --- |
  |  |  |
  | --- | --- |
  | main.ml2db.al |  |
  | test.ml2db.al |  |
  | test.ml2db.al.utility.confidence |  |
  | test.ml2db.al.utility.globaldistance |  |
  | test.ml2db.al.utility.neighbourhood |  |
  | test.ml2db.al.utility.pickers |  |
- - ### Uses of ExperimentDetails in main.ml2db.al

    Methods in main.ml2db.al that return ExperimentDetails

    | Modifier and Type | Method and Description |
    | --- | --- |
    |  |  |
    | --- | --- |
    | `ExperimentDetails` | ActiveLearning.`getExperimentDetails()` |

    Methods in main.ml2db.al with parameters of type ExperimentDetails

    | Modifier and Type | Method and Description |
    | --- | --- |
    |  |  |
    | --- | --- |
    | `void` | ActiveLearning.`initialise(ExperimentDetails experimentManager, int numberOfNeighbours, java.lang.String resultsDirectoryPath, int stepLimit)` |

    Constructors in main.ml2db.al with parameters of type ExperimentDetails

    Constructor and Description

    `ActiveLearning(ExperimentDetails experimentData,
    int numberOfNeighbours,
    java.lang.String resultsDirectoryPath)`

    `ActiveLearning(ExperimentDetails experimentData,
    int numberOfNeighbours,
    java.lang.String resultsDirectoryPath,
    int stepLimit)`
  - ### Uses of ExperimentDetails in test.ml2db.al

    Methods in test.ml2db.al that return ExperimentDetails

    | Modifier and Type | Method and Description |
    | --- | --- |
    |  |  |
    | --- | --- |
    | `static ExperimentDetails` | ExperimentDetailsTest.`generateExperimentManager(int unlabelledArffId, UtilityMetric metric)` |
  - ### Uses of ExperimentDetails in test.ml2db.al.utility.confidence

    Methods in test.ml2db.al.utility.confidence that return ExperimentDetails

    | Modifier and Type | Method and Description |
    | --- | --- |
    |  |  |
    | --- | --- |
    | `static ExperimentDetails` | ConfidenceMetricTest.`getExpManOnTestsetWithConfidenceMetric()` |
  - ### Uses of ExperimentDetails in test.ml2db.al.utility.globaldistance

    Methods in test.ml2db.al.utility.globaldistance that return ExperimentDetails

    | Modifier and Type | Method and Description |
    | --- | --- |
    |  |  |
    | --- | --- |
    | `static ExperimentDetails` | DistanceExperimentDetailsTest.`getExperimentManagerTestsetWithPositioning()` |
    | `static ExperimentDetails` | DistanceExperimentDetailsTest.`getExperimentManagerTrainsetWithPositioning()` |
    | `static ExperimentDetails` | DistanceExperimentDetailsTest.`getExpManTestsetWithGlobalPositAndConfidence()` |
  - ### Uses of ExperimentDetails in test.ml2db.al.utility.neighbourhood

    Methods in test.ml2db.al.utility.neighbourhood that return ExperimentDetails

    | Modifier and Type | Method and Description |
    | --- | --- |
    |  |  |
    | --- | --- |
    | `static ExperimentDetails` | NeighbourhoodExperimentDetailsTest.`getExperimentManagerTestset()` |
    | `static ExperimentDetails` | NeighbourhoodExperimentDetailsTest.`getExperimentManagerTrainset()` |
  - ### Uses of ExperimentDetails in test.ml2db.al.utility.pickers

    Methods in test.ml2db.al.utility.pickers that return ExperimentDetails

    | Modifier and Type | Method and Description |
    | --- | --- |
    |  |  |
    | --- | --- |
    | `static ExperimentDetails` | RandomAttributePickerTest.`getExpManagerTestsetWithRandomAttPicker()` |

- Overview
- Package
- Class
- Use
- Tree
- Deprecated
- Index
- Help

- Prev
- Next

- Frames
- No Frames

- All Classes
